# Prevalence and antimicrobial resistance profile of *Staphylococcus* in dairy farms, abattoir and humans in Addis Ababa, Ethiopia

**DOI:** 10.1186/s13104-017-2487-y

**Published:** 2017-04-28

**Authors:** Takele Beyene, Halefom Hayishe, Fikru Gizaw, Ashenafi Feyisa Beyi, Fufa Abunna, Bedaso Mammo, Dinka Ayana, Hika Waktole, Reta Duguma Abdi

**Affiliations:** 1 Department of Biomedical Sciences, College of Veterinary Medicine and Agriculture, Addis Ababa University, Bishoftu, Ethiopia; 2 Department of Pharmacology and Clinical Pharmacy, College of Health Sciences, Addis Ababa University, Addis Ababa, Ethiopia; 3 School of Veterinary Medicine, Samara University, Samara, Ethiopia; 4 Department of Animal Sciences, University of Florida, Gainesville, FL USA; 5 Department of Clinical Studies, College of Veterinary Medicine and Agriculture, Addis Ababa University, Bishoftu, Ethiopia; 6 Department of Microbiology, Immunology and Veterinary Public Health, College of Veterinary Medicine and Agriculture, Addis Ababa University, Bishoftu, Ethiopia; 7 Department of Veterinary parasitology and pathology, College of Veterinary Medicine and Agriculture, Addis Ababa University, Bishoftu, Ethiopia; 8 Department of Animal Sciences, University of Tennessee, Knoxville, TN USA

**Keywords:** Abattoir, Antimicrobial susceptibility, Dairy farms, Food safety, Humans, Multi-drug resistance, *Staphylococcus*, VRSA

## Abstract

**Background:**

*Staphylococcus* species cause mastitis and wound infection in livestock and food poisoning in humans through ingestion of contaminated foods, including meat and dairy products. They are evolving pathogens in that they readily acquire drug resistance, and multiple drug-resistant (MDR) isolates are increasing in human and veterinary healthcare. Therefore, this study was conducted to evaluate the prevalence of *Staphylococci* and their drug resistance in dairy farms and abattoir settings of Addis Ababa.

**Methods:**

In this cross-sectional study, 193 samples of milk, meat, equipment and humans working in the dairy farms and abattoir were collected (dairy farms = 72 and abattoir sources = 121). *Staphylococcus* isolation and identification at the species level was done according to ISO-6888-3 using biochemical characteristics. An antimicrobial susceptibility test was conducted for 43 of the isolates using 15 antimicrobial agents commonly used for humans and livestock by the Kirby Bauer disk diffusion method following CLSI guidelines.

**Results:**

*Staphylococcus* organism were isolated from 92 (47.7%) of the total 193 samples, 50% in the dairy farms and 46.3% in the abattoir. The isolated species were *S. aureus* (n = 31; 16.1%), *S. intermedius* (n = 21; 10.9%), *S. hyicus* (n = 16; 8.3%), and coagulase negative *Staphylococcus* (CNS) (n = 24; 12.4%). Gentamycin was effective drug as all isolates (n = 43; 100%) were susceptible to it and followed by kanamycin (n = 39; 90.7%). However, the majority of the isolates showed resistance to penicillin-G (95.3%), nalidixic acid (88.4%), cloxacillin (79.1%), vancomycin (65.1%) and cefoxitin (55.8%). Of the 15 *S. aureus* tested for drug susceptibility, 73.3% of them were phenotypically resistant to vancomycin (VRSA) and all of the 15 isolates showed multi-drug resistance (MDR) to >3 drugs. Also, all of the tested CNS (100%), *S. hyicus* (100%) and the majority of *S. intermedius* isolates (88.9%) developed MDR.

**Conclusion:**

Alarmingly, the *Staphylococcus* isolates circulating in the dairy farms and abattoir in the study area harbor MDR. High level of *Staphylococcus* species isolation from personnel and equipment besides food (meat and milk) samples in dairy farms and abattoir settings reveals that the hygiene practice in the dairy farm and abattoir is substandard. Prudent drug use and improved hygienic practice is recommended in the dairy farms and abattoir to safeguard the public from the risk of acquiring infections and MDR pathogenic *Staphylococcus*.

## Background

The genus *Staphylococcus* comprises of several species and subspecies [1]. The genus is broadly grouped into two, namely, coagulase- positive and coagulase-negative *Staphylococcus* (CNS) [2, 3]. CNS consists of a group of various *Staphylococcus* species that affect diverse host ranges. Some of them have evolved to cause mastitis in farm animals [4] whilst some species colonize post-surgical wounds (or cause infections in immunocompromised people) in humans [3]. Of the coagulase-positive *Staphylococci* group, the three major pathogenic ones are *S. aureus*, *S. intermedius* and *S. hyicus* [5, 6]. These three species are associated in many diverse and specialized forms with healthy and diseased farm animals and most of common pet animals [5]. They are the major pathogen causing mastitis although have tropism to different body parts of various hosts [5, 7, 8]. They are versatile pathogens with the ability to transfer between humans and livestock [6, 9, 10]. In human, they cause mild skin infection to more severe diseases such as pneumonia and septicemia [11]. In animals, their frequent involvement in mastitis results in contamination of milk and dairy products. They may found in a variety of locations in the dairy farms, abattoirs, food-processing units and on the body parts of the animals [12]. *Staphylococci* are also responsible for contamination of food products, food spoilage, reduction of food safety and shelf life and cause food-borne poisoning via production of deadly enterotoxins [13–17]. Infected individuals are the source of *Staphylococci* food contamination and intoxication at any point along the food chain [18]. *Staphylococcal* food poisoning has been reported as third cause of food-borne illnesses in the world [19].

Food is also an important vehicle for the transfer of antimicrobial resistant (AMR) factor to intestinal tract of consumers very efficiently [20, 21]. AMR has become the main public health burden worldwide [22]. Further transfer of AMR bacteria to humans via the food chain [23] and from livestock has been well documented [8]. In this context, livestock, milk and meat may serve as reservoirs for human infections by AMR *Staphylococcus* strains, thus allowing these microorganisms to persist and spread in the community. Up to now, many researchers have focused on the spread of resistant *S. aureus* in clinical setting [11, 24]. However, only handful of studies have been conducted about the presence of AMR in food animals in Ethiopia [25, 26] and moreover, *S. intermedius, S. hyicus* and CNS have been poorly studied. Multiple-drug resistant (MDR) staphylococcal isolates such as methicillin-resistant *S. aureus* (MRSA) have been isolated primarily from human, and also from animal samples [27, 28]. A study conducted by Schmidt et al. [29] in the KwaZulu-Natal province of South Africa have reported humans working with farm animals carry far more AMR *Staphylococci* than the farm animals they work with [29]. Thus, the transfer of AMR strains of *Staphylococcus* between humans and cows may result in serious health problems.

In Ethiopia, irrational antimicrobial use and misuse behavior is frequent [30], which could exacerbate the AMR issue in the country. The limited study on *Staphylococcus* species isolated from chicken-related samples in central and southern Ethiopia [31, 32] and *Salmonella* from cattle-related samples in south-eastern Ethiopia [33] have reported an alarming level of MDR in both bacteria. However, a good estimate of the magnitude of the prevalence of *Staphylococcus* species and their AMR pattern for both human and animals are hardly available in Ethiopia. Therefore, the aims of this study were to (i) isolate and identify *Staphylococcus* species in dairy farms and slaughterhouses in Addis Ababa via sampling animals, food (milk and meat), farm and abattoir equipment and personnel, (ii) determine their prevalence distribution within farm and abattoir in different sample types, and (iii) evaluate antimicrobial susceptibility profile of *Staphylococcus* species.

## Methods

### Study area and design

This cross-sectional study was conducted from October 2013 to April 2014 at Addis Ababa, which is situated at latitude of 9°3′ and 38°43′ East [34]. Samples were collected from randomly selected dairy farms (dairy cows, farm personnel and dairy equipment) and Addis Ababa abattoir enterprise (slaughtered cattle, abattoir personnel and abattoir materials). Hygienic practices of the pre-milking, milking and post milking activities of dairy farms and pre-slaughtering, slaughtering and post slaughtering activities were assessed using a structured questionnaire.

### Sample types, collection, transportation and storage

Overall, 193 samples were collected; samples from dairy farms included raw pooled udder milk (n = 40), tank milk (n = 8), pooled tank swabs (n = 8), pooled bucket swabs (n = 8) and pooled milkier hand swabs (n = 8). From the abattoir carcass swabs (n = 103), pooled slaughter lines swab from hanging materials (n = 6), pooled knife swabs (n = 6), pooled hand swabs (n = 6) were collected.

Samples were collected aseptically from each critical point. Raw milk samples were collected from two critical points, from the cows’ udder and milking bucket together with milkiers’ hand and container swabs. The swabbing was done before milk sampling using sterile swabs. From the abattoir, a pooled carcass swab included neck, forelegs, thoracic, abdomen, and hind legs. In addition, the slaughter personnel’s hands, surface of meat-cutting equipment and slaughter line were swabbed [35] and each swab was kept in a different material.

For every swab sample, a sterile single screw capped tube filled with 4 ml of buffered peptone water (BPW) was used. Prior to sampling, swab tips were moistened in the BPW and during sampling, the swab was rotated and rubbed against the sampled surface several times. After completion of swabbing, the swab was placed inside a screw-capped tube containing BPW and transported using a cold chain to Microbiology laboratory of College of Veterinary Medicine and Agriculture, Addis Ababa University and stored at +4 °C for a maximum of 24 h until it was cultured [35].

### Study methodology

#### Bacterial culture and species identification

The techniques recommended by the International Organization for Standardization, ISO 6888-3: 2003 [35] was employed for the isolation and identification of *Staphylococcus* species from abattoir and farm samples. Final identification of *Staphylococci* organisms and species assignment were done based on Gram staining, hemolysis of blood agar, catalase test, sugar fermentation test, oxidase test, coagulase test, oxidation–fermentation test, and growth on mannitol salt agar and Purple agar base [35]. The biochemical test types used and the responses of *Staphylococci* species to the biochemical tests were summarized as shown in Table 1.

**Table 1:** Differential tests used for identification of *Staphylococcus* species and their reactions

*Staphylococcus* species	Hemolysis	Pigment production	Gram stain	Coagulase test	Catalase test	OF test	Fermentation of sugar		Susceptibility to polymyxin B (300 unit)
							MSA	PAB	
*S. aureus*	+	+	+	+	+	F	+	+	R
*S. intermidius*	+	–	+	+	+	F	±	±	S
*S. hyicus*	–	–	+	+	+	F	–	–	R
CNS	–	–	+	–	+	F	–	–	NP

+, 90% or more strains are positive; ±, 90% or more strains are weakly positive; −, 90% or more strains are negative

*CNS* coagulase negative *staphylococcus*, *F* fermentative, *OF* oxidation–fermentation, *MSA* mannitol salt agar, *PAB* Purple agar base, *R* resistant, *S* susceptible, *NP* not performed

#### Antimicrobial sensitivity test

Disc diffusion testing was performed for *Staphylococcal* isolates in accordance with the Clinical and Laboratory Standards Institute (CLSI) (2008) [36]. One representative antimicrobial agent from each subclass of commonly used antimicrobials for treatment of bovine mastitis or considered as important antimicrobial agents for human was selected for susceptibility testing. The phenotypic antimicrobial sensitivity response of each isolate was evaluated using panel of 15 antimicrobial discs. The used antimicrobial agents included 15 μg erythromycin (E), 10 μg streptomycin (S), 50 μg nitrofurantoin (F), 10 unit penicillin-G (P), 5 μg ciprofloxacin (CIP), 25 μg sulfamethoxazole-trimethoprim (SXT), 5 μg cloxacillin (OB), 30 μg cefoxitin (FOX), 30 μg chloramphenicol (C), 30 μg tetracycline (TE), 10 μg gentamycin (CN), 30 μg vancomycin (VA), 30 μg kanamycin (K), 30 μg amoxicillin (AML), and 30 μg nalidixic acid (NA). All the discs were purchased from Oxoid, UK. The diameters of the zone of inhibition around the disks were measured to the nearest millimeter using rulers. The isolates were classified as susceptible, intermediate and resistant according to the interpretative standards of CLSI (2008) [36]. Isolates showing resistance to three or more antimicrobial subclass were considered as multiple drug resistant (MDR) [37].

### Data analysis

Data was analyzed using SPSS software package (SPSS 20.0 for window 7, SPSS Inc, Chicago, Illinois). The proportions of *Staphylococcus* isolates in samples from the abattoir and dairy farms were computed. The Chi square (χ^2^) test was used to determine the difference in proportions of *Staphylococcus* among different sample types and sources. The significance level of α = 0.05 was taken as a cut-off value.

## Results

Our personal observation during sample collection showed that dairy farms and abattoir workers practiced substandard hygiene. The number of *Staphylococcus* species isolated from personnel and equipment in dairy farms and abattoir was presented in Table 2.

**Table 2:** Proportions of *Staphylococcus* species isolated (n = 92) from dairy farms and abattoirs in Addis Ababa, Ethiopia

Sample source	Sample type	*S. aureus* N (%)	*S. intermedius* N (%)	*S. hyicus* N (%)	CNS N (%)	Total N (%)
Abattoir	AHS	0 (0.0)	0 (0.0)	0 (0.0)	1 (16.7)	1 (16.7)
	KS	2 (33.3)	2 (33.3)	1 (16.7)	0 (0.0)	5 (83.3)
	SLS	2 (33.3)	0 (0.0)	0 (0.0)	3 (50.0)	5 (83.3)
	CS	12 (11.7)	13 (12.6)	9 (8.7)	11 (10.7)	45 (43.7)
Farm	BS	2 (25.0)	0 (0.0)	0 (0.0)	1 (12.5)	3 (37.5)
	FHS	2 (25.0)	1 (12.5)	1 (12.5)	1 (12.5)	5 (62.5)
	TM	2 (25.0)	0 (0.0)	1 (12.5)	1 (12.5)	4 (50.0)
	TS	1 (12.5)	1 (12.5)	0 (0.0)	2 (25.0)	4 (50.0)
	UM	8 (20.0)	4 (10.0)	4 (10.0)	4 (10.0)	20 (50.0)
	Total	31 (16.1)	21 (10.9)	16 (8.3)	24 (12.4)	92 (47.7)
	χ^2^ (P value)	7.2 (0.5)	6.9 (0.5)	3.6 (0.9)	10.4 (0.2)	10.2 (0.2)

*AHS* hand swab from abattoir, *CNS* coagulase negative *Staphylococcus*, *KS* knives swab, *SLS* slaughter line swab, *CS* carcass swab, *BS* bucket swab, *FHS* hand swabs from farm, *TM* tanks milk, *TS* tanks swab, *UM* udder milk, *N* number

Out of 193 samples, *Staphylococci* were isolated from 92 (47.7%) samples, 36 from the dairy farms and 56 from the abattoir. The proportion of *Staphylococcus* isolates in the sample sources and types varied, but not significant (P > 0.05). Based on the biochemical tests, the predominant species was *S. aureus* (n = 31; 16.1%), followed by CNS (n = 24; 12.4%), *S. intermedius* (n = 21; 10.9%) and *S. hyicus* (n = 16; 8.3%) (Table 2).

### *Staphylococcus* isolates from dairy farm

The 36 (50.0%) isolates identified as *Staphylococci* species from dairy settings were tested for species assignment using biochemical characteristics. The majority of them were *S. aureus* (n = 15; 20.8%), followed by CNS (n = 9; 12.5%), *S. intermedius* (n = 6; 8.3%) and *S. hyicus* (n = 6; 8.3%). The frequency of isolation of *Staphylococcus* varied between sample types and ranged from 37.5 to 62.5%. Highest prevalence was observed in milkier hand swabs (62.5%), however, the difference was not significant (P > 0.05) (Table 2).

### *Staphylococcus* isolates from abattoir

As indicated above from 121 abattoir source samples, 56 (46.3%) isolates of *Staphylococcus* species were isolated. These isolates were grouped by biochemical test into *S. aureus* (n = 16; 13.2%), CNS (n = 15; 12.4%), *S. intermedius* (n = 15; 12.4%), and *S. hyicus* (n = 10; 8.3%). The frequency of isolation of *Staphylococcus* varied between sample types and ranged from 16.7 to 83.3%. The results showed a comparatively higher prevalence in knife and slaughter hanging material swabs, each with 83.3%. This difference was statistically significant (P < 0.05).

### *Staphylococcus* isolates from personnel

*Staphylococcus* was isolated from 14 hand swabs from dairy farm workers (n = 8) and abattoir workers (n = 6). *Staphylococcus* species was isolated from six (42.9%) hand swab samples. The six isolates identified using biochemical characteristics as *S. aureus* (n = 2; 14.3%), CNS (n = 2; 14.3%), *S. intermedius* (n = 1; 7.1%), and *S. hyicus* (n = 1; 7.1%). The results showed a relatively higher prevalence in farm milkier hand swab having five (83.3%) compared to the one isolate (16.7%) from abattoir workers’ hand swab. However, the difference was not statistically significant (P > 0.05).

### Antimicrobial susceptibility testing

Overall, antimicrobial susceptibility test was performed on 43 *Staphylococcus* isolates. Eighteen (41.9%) isolates were from farms and 25 (58.1%) from abattoir. The 43 isolates tested comprised of *S. aureus* (n = 15; 34.9%), CNS (n = 13; 30.2%), *S. intermedius* (n = 9; 20.9%), and *S. hyicus (*n = 6; 14.0%). Penicillin-G (P) resistance was the highest widespread resistance recorded among the isolates in this study (n = 41 isolates; 95.3%), followed by NA 38 (88.4%), OB 34 (79.1%), VA 28 (65.1%) and FOX 24 (55.8%). Conversely, lower resistance was recorded against K with 39 (90.7%) susceptible and four (9.3%) intermediates (Table 3). Of the 15 antibiotics tested, CN was currently the most effective drug as all of the 43 (100.0%) isolates were susceptible. Among the isolates resistant to VA, *S. aureus* and CNS contributed for 39.3% (11/28) and 32.1% (9/28), respectively.

**Table 3:** Antimicrobial susceptibility profile of *Staphylococcus* isolates (n = 43) from dairy farms and abattoir in Addis Ababa, Ethiopia

Drug	Susceptible N (%)	Intermediate N (%)	Resistant N (%)
Amoxicillin (AML)	33 (76.7)	0 (0.0)	10 (23.3)
Cefoxitin (FOX)	19 (44.2)	0 (0.0)	24 (55.8)
Chloramphenicol (C)	27 (62.8)	6 (14.0)	10 (23.3)
Ciprofloxacin (CIP)	37 (85.0)	5 (11.6)	1 (2.3)
Cloxacillin (OB)	9 (20.9)	0 (0.0)	34 (79.1)
Erythromycin (E)	14 (32.6)	9 (20.9)	20 (46.5)
Gentamicin (CN)	43 (100.0)	0 (0.0)	0 (0.0)
Kanamycin (K)	39 (90.7)	4 (9.3)	0 (0.0)
Nalidixic acid (NA)	4 (9.3)	1(2.3)	38 (88.4)
Nitrofurantoin (F)	14 (32.6)	8 (18.6)	21 (48.8)
Penicillin-G (P)	2 (4.7)	0 (0.0)	41 (95.3)
Streptomycin (S)	29 (67.4)	7 (16.3)	7 (16.3)
Sulfamethoxazole-trimethoprim (SXT)	33 (76.7)	1 (2.3)	9 (20.9)
Tetracycline (TE)	26 (60.5)	2 (4.6)	15 (34.9)
Vancomycin (VA)	15 (34.9)	0 (0.0)	28 (65.1)

Key: *N* number

In this study, all of the *S. aureus* (n = 15; 100%), CNS (n = 13; 100%) and *S. hyicus* (n = 6; 100%) isolates exhibited MDR as each isolate at least resistant to >3 drugs simultaneously. Among the nine *S. intermedius* isolates tested, 8 (88.9%) of them exhibited MDR. Alarmingly, some isolates of *Staphylococcus* species demonstrated resistance to 13 different antimicrobial subclasses (MDR) at a time as shown in Table 4. The comparison of AMR patterns among various sample types showed there was not statistically significant differences (P > 0.05).

**Table 4:** Multiple drug-resistance profile of *Staphylococci* isolates

Number of drugs showing resistance	Frequency	Number and % of				Total %
		*S. aureus*	*S. intermedius*	*S. hyicus*	CNS	
0	1	0 (0.0)	1 (11.1)	0 (0.0)	0 (0.0)	2.3
3	3	1 (6.7)	1 (11.1)	1 (16.7)	0 (0.0)	7.0
4	5	3 (20.0)	1 (11.1)	0 (0.0)	1 (7.7)	11.6
5	9	4 (26.7)	2 (22.2)	2 (33.3)	1 (7.7)	20.9
6	8	2 (13.3)	2 (22.2)	1 (16.7)	3 (23.1)	18.6
7	7	2 (13.3)	0 (0.0)	1 (16.7)	4 (30.8)	16.3
8	5	2 (13.3)	1 (11.1)	0 (0.0)	2 (15.4)	11.6
9	3	1 (6.7)	0 (0.0)	1 (16.7)	1 (7.7)	7.0
10	1	0 (0.0)	0 (0.0)	0 (0.0)	1 (7.7)	2.3
13	1	0 (0.0)	1 (11.1)	0 (0.0)	0 (0.0)	2.3
Total	43	15 (100)	9 (100)	6 (100)	13 (100)	100
% with MDR		15 (34.9)	8 (18.6)	6 (14.0)	13 (30.2)	97.7 (100)
χ^2^ (P value)		3.89 (0.91)	10.84 (0.287)	4.51 (0.875)	9.13 (0.426)	

*CNS* coagulase negative *Staphylococcus*, *MDR* multiple drug-resistance

## Discussion

Both coagulase-positive *Staphylococci* and CNS are commensal opportunistic infections of animals and humans [38]. They cause superficial skin infection, mastitis and severe invasive diseases in farm animals and wound infections in humans [3, 5]. They are also well known for their food poisoning via their enterotoxins [9, 13–17] and for their ability to acquire AMR [38]. However, a good estimate of the magnitude of the prevalence of *Staphylococcus* species and their AMR pattern in different livestock production settings, in foods of livestock origin, and in humans involved in handling of the foods are poorly available in Ethiopia. Therefore, this study was conducted to evaluate the prevalence of *Staphylococci* and their drug resistance in dairy farms and abattoir settings of Addis Ababa.

In this study, the overall prevalence of *Staphylococci* was 47.7% of 193 tested samples collected from dairy farm and abattoir settings, suggesting the significant association of *Staphylococci* species with livestock. The *Staphylococci* prevalence was higher at both dairy farm (50.0%) and abattoir (46.3%) settings. The substandard hygienic practices during pre-milking, milking and post milking activities in dairy farms might have contributed. Milk hygiene and udder-cleaning operation was insufficiently conducted in Ethiopia triggering flare up of mastitis [39]. In this regard, poor pre-milking udder preparations could play an important part in the contamination of milk during milking [40, 41]. Similarly, poor hygienic practice was noticed in pre-slaughtering, slaughtering and post slaughtering activities within the abattoir during this study. In this regards, 15.4% of the abattoir workers had no health certificate and 11.3% of the butchers did not use protective clothes. There was no hot water, sterilizer and cooling facility in the abattoir in Ethiopia [42].

The higher prevalence of *Staphylococci* at both dairy farm and abattoir settings in this study, indicating its (i) serious economic, animal welfare, food safety and public health problem and (ii) association to livestock for its transmission between animals and humans in livestock farm setting. Similar findings in its association with livestock have been reported from elsewhere [43, 44]. The predominant *Staphylococci* species detected in this study were *S. aureus* (16.1%), followed by CNS (12.4%), *S. intermedius* (10.9%), and *S. hyicus* (8.3%). These four *Staphylococci* species group are said to be the most pathogenic [5]. Furthermore, some animal-associated clones within these species group appear to have the capacity to colonize and cause disease in multiple host species [45]. They are versatile pathogens with the ability to transfer between humans and different livestock [6, 9, 10] with a possible switch from one-to-another host species [44, 46].

The *Staphylococci* species resilience characteristics might be revealed in their ability to appear in a variety of locations in this study. In this regard, we isolated them from bucket swab (37.5%), tanks swab (50.0%), tanks milk (50.0%), udder milk (50.0%), and milkier hand swabs (62.5%) in diary settings. We also isolated them from carcass meat (43.7%), abattoir workers’ hand (16.7%), knife (83.3%) and slaughter hanging material (83.3%) in abattoir settings. Interestingly, 16.7% of abattoir workers and 83.3% of milkier personnel carry *Staphylococci* in their hand. It has been reported that about one-third of humans carry *Staphylococci* in their nasal cavity [46]. Thus, frequently washing of the abattoir slaughterers hand could be the possible factor responsible for the relatively low prevalence of *Staphylococcus* in abattoir. In this study, *Staphylococcus* isolates were isolated more from carcass meat swab from slaughtered cattle than any other samples. This could be attributed to the higher sample size of the carcass meat samples than others. The higher number of personnel workers at abattoir might have also contributed to carcass contamination. In general, our result revealed the substandard milk and meat hygiene and personnel and equipment hygiene practices in dairy farms and abattoirs during handling, storage and processing of foods of livestock origin. Our finding agrees with the previous report from dairy farms, slaughterhouses, food-processing units and on the body parts of the animals and humans [12, 47]. For example, *S. aureus* (13.2%), CNS (12.4%), *S. intermedius* (12.4%), and *S. hyicus* (8.3%) were isolated from abattoir including carcass meat. Isolation of these *Staphylococci* species from meat demonstrated that they have entered the food chain to affect the food safety and public health [48, 49]. Contamination of milk and meat by them could lead to food poisoning through production of *Staphylococcal* enterotoxins. Available literature indicated that 62.9% of isolates from sheep milk [50] and 15–55.9% of isolates from dairy products [16, 51–53] produce enterotoxin with marked geographical differences. Although this is beyond the scope of our paper, the culture of raw milk and raw meat consumption in Ethiopia might be potential sources of *Staphylococcal* enterotoxin food poisonings to the public.

The prevalence of *S. aureus* in 193 tested samples was 31(16.1%). *S. aureus* was isolated from nearly all sample types with different proportion, but not from abattoir slaughter worker’s hand swab (0.0%). This finding is similar with the 17.2% prevalence in Egypt [54] and 19.5% from human and animal sources [55], but lower than 75% in bovine bulk milks [56], 68% [14], 61.3% [57], 40% [58] and 82.2% from cheeses made from raw cow’s milk [59]. The 10.9% *S. intermedius* prevalence detected in our study was similar with the 13% in cheese samples [59] but higher than the 2% in milk and milk product samples [58]. Similarly, the prevalence of *S. hyicus* was 8.3% in our study. Almost half of the total isolates were isolated from carcass swab. It was higher than the 4% in milk and milk product samples [58] and 4.5% in cheeses [59]. CNS was the second most isolated species next to *S. aureus* in this study. It was isolated from almost all the samples except in knife swab. The predominant proportion of CNS was isolated from carcass swab. The overall prevalence of CNS was 12.4%, which was lower than the 54% in raw milk of cattle in Mongolia [60] and 29% [59]. The high number of CNS isolation in the current study may be indicator for poor hygienic practices in the dairy farms and abattoir. CNS is a part of the normal teat skin flora, mucosa of humans and animals, and also found free living in the environment [61, 62]. In the past, CNS was often regarded as skin flora opportunists. However, recent data indicate that they are associated with several subclinical and clinical infections [63].

Of 43 isolates, 42 (97.7%) had resistance to at least one of the 15 antimicrobials tested. It had been reported that about 90% of *S. aureus* isolated from patients was found resistant in 1980s [64]. The antimicrobials we currently evaluated could be subjectively classified into four based on magnitude of resistance recorded in this study. These include (i) highly effective antimicrobials where all isolates were susceptible, (ii) effective antimicrobials as >95% isolates were susceptible, (iii) still usable antimicrobials as >50% of isolates were susceptible, and (iv) not recommended for use as >55% of the isolates were resistant. In this regard, the general drug use policy recommends antimicrobials for use especially for treatment of infection in humans only if less than half (<55%) of the isolates are resistant [65].

Accordingly, CN and K are the most effective antimicrobials as all of the 43 *Staphylococci* isolates were susceptible to both in this study. In fact, four (9.3%) of the 43 isolates had intermediate breakpoint response to K. Our finding was similar to earlier studies in other countries [55, 66, 67]. Few isolates had resistance to CIP (2.3%), thus, fits to the second antimicrobial category listed above. Third, resistant among isolates ranges between 16.3 and 48.8% for some antimicrobials in this study. Antimicrobials in this group include S (16.3%), SXT (20.9%), AML (23.3%), C (23.3%), TE (34.9%), E (46.5%), and F (48.8%). Our finding is comparable to the findings of others [26], but contrary to the findings in Taiwan [47]. Fourth, >55% of the *Staphylococci* isolates had resistance to cefoxitin (55.8%), VA (65.1%), OB (79.1%), NA (88.4%), and P (95.3%). The resistance value recorded to P (95.3%) was the highest in this study. In another study in different geographical areas showed that it was 87.2% in Ethiopia [68], 80% in Sweden [69], 57% in Iran [70], 50% in Finland [71], and 23% in West India [72]. In a separate study, 70–73% of *S. aureus* strains isolated from various foods were resistant to β-lactam such as P and ampicillin [73]. Penicillin (P) and other β-lactam antibiotics are the most commonly used antimicrobials for the treatment of infection of mastitis in veterinary practice in Ethiopia [64]. The higher prevalence of penicillin resistant isolate could be, therefore, related to prolonged, improper and indiscriminate usage as suggested by some authors [64, 74].

Overall, all (100%) of *S. aureus*, CNS, and *S. hyicus* (whilst 88.9% of *S. intermedius*) isolates had resistance to >3 antimicrobials (MDR) of all the tested drugs. The share of MDR isolate by *Staphylococci* species among 43 isolates was 34.9% *S. aureus*, 30.2% CNS, 20.9% *S. intermedius*, and 14.0% *S. hyicus* (Table 3) although it was not significantly different between *Staphylococcus* species. Most of MDR was primarily due to P, NA, OB, VA and FOX in this study. This finding is higher than the study result of Moon et al. [75] who reported that about two out of 21 *S. aureus* has been reported to be resistant to >3 of non-β-lactam antimicrobials including P and E [75]. Furthermore, higher MDR was observed in our study than (79%) among isolates reported in Korea [76] and at Jimma Hospital, Ethiopia (80%) [77]. The abundance of MDR *Staphylococci* isolated from milk (farm), meat (abattoir), equipment and humans could pose a risk to public health. Transfer of antimicrobial resistant bacteria to humans has been reported via the food chain [23] and from livestock [8]. AMR is a major public health burden worldwide [22]. *Staphylococcus* strains have developed MDR worldwide with broad diversity in prevalence rate in different regions [37].

## Conclusions

The present study revealed high proportion of *Staphylococcus* species (47.7%) (*S. aureus, S. intermedius, S. hyicus* and CNS) in dairy farms and abattoir settings at Addis Ababa. Higher proportion of isolation rate of *Staphylococcus* species from farm and abattoir personnel signals the existence of poor hygienic practices and consequently, its public health implication. All the tested isolates for antimicrobial susceptibility showed resistance to at least three antimicrobial agents except one *S. intermidius* isolate susceptible to all tested drugs. The isolates also showed good susceptibility profile to CN, K, and CIP, but resistance was observed against P, NA, OB, VA and FOX. Moreover, MDR was also observed against some of the commonly used antimicrobial agents. Thus, improving hygienic measures in the dairy farms as well as within the abattoir must be effected to safeguard the public from risk of staphylococcal food poisoning and acquiring MDR isolates. Additionally, future research should consider identifying *Staphylococcus* enterotoxins from milk and meat and genes responsible for AMR in Ethiopia.

## Abbreviations

AML: amoxicillin
AMR: antimicrobial resistance
BAP: blood agar plates
BPW: buffer peptone water
C: chloramphenicol
CFU: colony forming units
CI: confidence interval
CIP: ciprofloxacin
CN: gentamycin
CNS: coagulase-negative *Staphylococcus*
E: erythromycin
ELFA: enzyme-linked fluorescent assay
F: nitrofurantoin
FOX: cefoxitin
ISO: International standard organization
K: kanamycin
MSA: mannitol salt agar
µm: micrometer
MDR: multidrug resistance
NA: nalidixic acid
NaCl: sodium chloride
NAP: nutrient agar plates
OB: cloxacillin
P: penicillin-G
PAB: Purple agar base
S: streptomycin
SEs: *Staphylococcal* enterotoxins
SXT: sulfamethoxazole-trimethoprim
TE: tetracycline
TSB: tryptone soya broth
VA: vancomycin
VRSA: vancomycin resistant *Staphylococcus aureus*
